# Development and external evaluation of an interpretable machine-learning model for early prediction of organ failure in higher-risk acute pancreatitis patients: A multicentre cohort study

**DOI:** 10.1371/journal.pdig.0001735

**Published:** 2026-09-22

**Authors:** Di Wu, Wenhao Cai, Chunmei Chen, Minting Chen, Yang Lv, Yilin Huang, Anthony Evans, Juan Lin, Diane Latawiec, Arjun Kattakayam, Rajarshi Mukherjee, Wei Huang, Qing Xia, Jie Xiao, Chunqiu Su, Jie Peng, Kuirong Jiang, Robert Sutton

**Affiliations:** 1 Pancreas Center, First Affiliated Hospital with Nanjing Medical University, Nanjing, Jiangsu, China; 2 Department of Geriatric Gastroenterology, Xiangya Hospital, Central South University, Changsha, Hunan, China; 3 Institute of Systems, Molecular and Integrative Biology, University of Liverpool, Liverpool, United Kingdom; 4 Emergency Department, the Third Xiangya Hospital, Central South University, Changsha, Hunan, China; 5 West China Centre of Excellence for Pancreatitis, Institute of Integrated Traditional Chinese and Western Medicine, West China-Liverpool Biomedical Research Centre, Chengdu, Sichuan, China; 6 Pediatric Hematology Laboratory, Division of Hematology/Oncology, Department of Pediatrics, The Seventh Affiliated Hospital of Sun Yat-Sen University, Shenzhen, Guangdong, China; 7 Department of Ophthalmology, Nanfang Hospital, Southern Medical University, Guangzhou, Guangdong, China; 8 Department of Information Center, The Affiliated Jiangning Hospital of Nanjing Medical University, Nanjing, Jiangsu, China; 9 Computational Biology Facility, University of Liverpool, Liverpool, United Kingdom; 10 Department of Radiology, First Affiliated Hospital with Nanjing Medical University, Nanjing Medical University, Nanjing, Jiangsu, China; Xiangtan Central Hospital, CHINA

## Abstract

Organ failure (OF) is the most important determinant of prognosis in acute pancreatitis (AP) and its duration defines disease severity. Early identification of individuals at high risk of developing OF is crucial. We aimed to develop machine-learning models, benchmarked against a feedforward multilayer-perceptron (MLP) neural network, to predict new-onset OF at admission using static admission-day variables. In this study, data were extracted from MIMIC-IV (development cohort) and a multicentre AP cohort from two Chinese tertiary teaching hospitals (evaluation cohort), which excluded mild AP and therefore comprised patients requiring ICU-level or high-dependency care. Features were selected with Boruta and LASSO. Five machine-learning models and one multilayer-perceptron benchmark were developed in MIMIC-IV and externally evaluated in the Xiangya cohort without updating. SHapley Additive exPlanations (SHAP) were used to visualize decision-making patterns and individual prediction interpretations. The best-performing model was deployed as an interactive, web-based tool. We found that in the discovery cohort, 341 (23.9%) of 1429 AP patients developed new-onset OF within 28 days of admission while 45 of 216 patients (20.8%) in the validation cohort developed OF. Boruta and LASSO algorithms identified six key predictors including blood urea nitrogen, platelets, triglyceride-glucose index, albumin, white blood cells, and partial thromboplastin time, which were used to construct the ML and MLP models. XGBoost gave the best discrimination on external evaluation (AUC 0.837, 95% CI 0.771–0.902). Logistic recalibration improved calibration, and the recalibrated XGBoost model was implemented as a web-based research prototype (Decent app). In conclusion, XGBoost predicted new-onset OF with good discrimination in a severity-enriched AP cohort, and SHAP made individual predictions interpretable. Model selection, threshold selection and recalibration all used the evaluation cohort, so the deployed model still requires an independent cohort. Prospective usability and clinical-impact studies are needed before clinical use.

## Author summary

Acute pancreatitis (AP) is a serious inflammatory condition where the development of organ failure (OF) is the most critical factor determining patient outcomes. Early identification of patients at high risk for OF is essential for improving care, but accurate prediction at the time of hospital admission remains a challenge. In this study, we developed machine-learning models, benchmarked against a multilayer perceptron, and evaluated them externally to predict new-onset OF in AP patients on admission to hospital. Using data from large international cohorts, we identified six key clinical characteristics at admission, including blood urea nitrogen, platelets, triglyceride-glucose index, albumin, white blood cells, and partial thromboplastin time levels, to train our models. Among the algorithms tested on admission-day tabular data, XGBoost performed best. The cohort used for evaluation excluded mild AP, so our results apply to patients needing ICU-level, high-dependency or close monitoring, not to unselected admissions. We then recalibrated the XGBoost model in the same cohort and built a web application (Decent app) that returns a risk estimate with an explanation of the factors behind it. Because the recalibration and the risk threshold came from the cohort in which we assessed them, the application has not been independently validated. It is a research prototype for risk calculation rather than a decision-support tool, and prospective usability, workflow and clinical-impact studies are needed first.

## Introduction

Acute pancreatitis (AP) is one of the most challenging and common gastroenterological diseases imposing an annual financial burden exceeding 2.6 billion dollars in the US [1]. According to both the Revised Atlanta Classification and Determinant-Based Classification, the presence of organ failure (OF) is a key determinant of AP severity and is strongly associated with early phase morbidity and mortality [2–4]. OF, as a generic term, is defined as significant functional impairment of an organ system that is critical to sustenance of life. In the context of AP, three major systems are considered most important: respiratory, renal, and cardiovascular [4]. From triage and prognostic perspectives, early prediction of new-onset OF in AP patients during initial presentation is critical. Timely risk stratification of high-risk patients is essential for optimizing clinical outcomes [5].

In recent years, several prediction scores and biochemical markers have been developed, and no single laboratory test is consistently accurate for the prediction of AP severity [6]. Commonly used scoring systems such as Acute Physiology and Chronic Health Evaluation II (APACHE II) and Bedside Index of Severity in Acute Pancreatitis (BISAP) are multifactorial and somewhat tedious to use [2]. Significant advances have been made in identifying serum biomarkers to stratify an early risk of the severe form of AP patients [7]. While BISAP or single prognostic marker, such as C-reactive protein (CRP), demonstrate sensitivity, they provide better severity prediction at 48 hours but still lack sufficient diagnostic accuracy within 24 hours [8]. Insulin resistance (IR) refers to the reduced sensitivity of insulin-dependent organs and tissues to both endogenous and exogenous insulin which is a significant risk factor for the development of cardiovascular failure [9]. The triglyceride-glucose (TyG) index, a simple and reliable surrogate for IR, has emerged as a robust prognostic predictor for cardiovascular failure, owing to its straightforward calculation and precision [9]. Emerging evidence indicates that elevated TyG index levels may also be associated with acute kidney injury in AP patients, however, its role in predicting OF in AP patients remain unexplored [10].

In previous studies, some traditional models have identified predictors of OF and mortality in AP patients, however the accuracy and specificity of these predictors remain suboptimal [11,12]. There is a lack of accurate prognostic tools for AP which could aid clinical decision-making and advance personalized medicine. AI-driven approaches can capture variable interactions and non-linear relationships that traditional modelling methods may miss [11–13]. Increasingly, ML techniques are being used to develop high-performing prognostic models in AP, however a recent systematic review revealed methodologic and reporting quality was poor, and most studies displayed reporting omissions, particularly regarding code sharing and human-AI interface design which are critical for model validity, reproducibility, and clinical implementation [14,15]. The validation of prediction models deserves more recognition facilitated by the provision of open-access tools [16]. Few studies have benchmarked tree-based machine learning against a neural network for OF prediction in AP using admission-day tabular data, and whether the neural network offers any advantage in this setting is unclear.

Our principal aims were 1) to develop machine-learning models with a multilayer-perceptron benchmark for new-onset OF prediction, and to evaluate them externally in a severity-enriched multicentre cohort; 2) to compare the candidate models in the evaluation cohort and select one for deployment, recognizing that this selection was not independent of that cohort; 3) to enhance model interpretability using SHapley Additive exPlanations (SHAP), with particular focus on the prognostic contribution of TyG index and 4) to make the selected model available as a web-based research prototype for risk calculation, which would require prospective evaluation before any clinical use.

## Materials and methods

### Ethics statement

As the MIMIC database is de-identified and retrospective, it was not necessary to obtain informed consent from patients. We made a verbal registration to our hospital’s ethics committee and passed the Collaborative Institutional Training Initiative program exam. As this is a retrospective cohort study, our study will not interfere with the enrolled patients. Institutional Review Board of Xiangya Hospital (No.202103047) and the Third Xiangya Hospital (No. 21019) approved the waiver of patient informed consent. This validation dataset and all methods have been performed in accordance with the Declaration of Helsinki. Information was gathered from electrical medical system in an anonymous manner. The authors ensured patient data confidentiality. The TRIPOD+AI reporting details are attached in the S1 Table.

### Data source, extraction and participants

**Fig 1:**
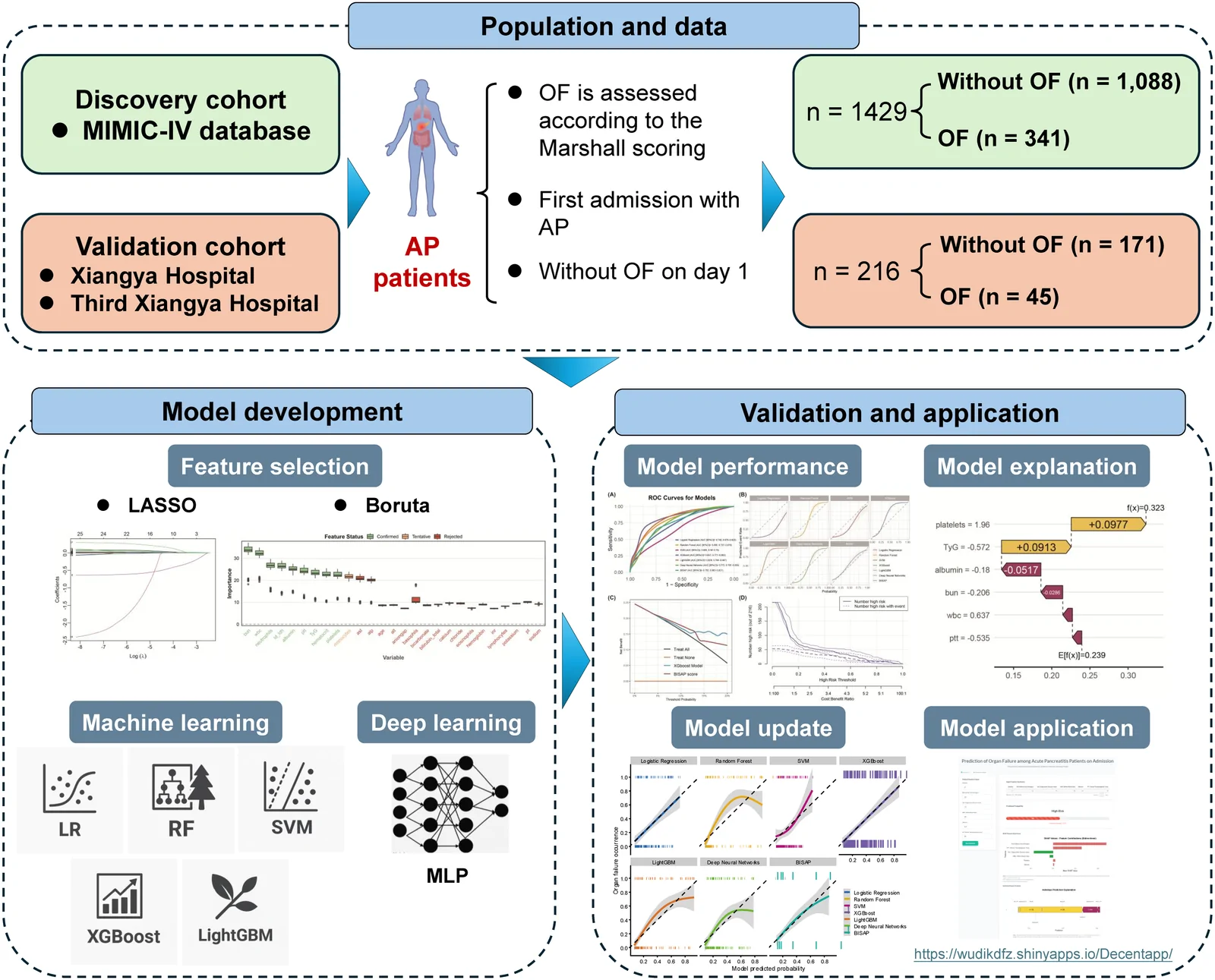
Overview of the study workflow. Abbreviations: MIMIC, Medical Information Mart for Intensive Care; OF, Organ Failure; ML, Machine Learning; LASSO, Least Absolute Shrinkage and Selection Operator; LR, Logistic Regression; RF, Random Forest; SVM, Support Vector Machine; XGBoost, eXtreme Gradient Boosting; LightGBM, Light Gradient Boosting Machine; MLP, Multilayer Perceptron; ROC, Receiver Operating Characteristic; SHAP, SHapley Additive exPlanations.

Databases used the Xiangya pancreatitis retrospective cohort database (2021–2024), sourced from both Xiangya hospital and the third Xiangya hospital, affiliated to Central South University, Changsha, China and the Medical Information Mart for Intensive Care IV (MIMIC-IV) database v3.1, which contains de-identified records of emergency department, hospitalization and ICU admissions at Beth Israel Deaconess Medical Center (2008–2022) [17]. Inclusion criteria: Patients with a diagnosis of AP were identified using International Classification of Diseases, Ninth/Tenth Revision (ICD-9/10) codes (ICD-9: 577.0; ICD-10: K85) from the MIMIC-IV database. Only patients with available clinical data within the first 24 hours of admission and documented outcomes up to 28 days were included. Exclusion criteria: (1) Patients with pre-existing OF at admission; (2) Patients with chronic pancreatitis or pancreatitis secondary to malignancy; (3) Age < 18 years. Waived informed consent was applied per MIMIC-IV’s de-identification protocol. The validation dataset, multi-center database in Xiangya hospital and Third Xiangya hospital, was approved by the Institutional Review Board of both Xiangya Hospital (No. 202103047) and the Third Xiangya hospital (No. 21019). Data from MIMIC-IV was extracted using PostgreSQL. OF is assessed according to the Marshall scoring system. This evaluates dysfunction with the total score determined by the highest individual score from the three organ systems: respiratory, cardiovascular, and renal that develops within 28 days after the first 24h from admission in both the discovery and validation cohorts [4]. The clinical characteristics during the first 24h and the following outcomes of OF within 28 days from admission were collected. Mild AP patients will be recovering within 7 days which could be a potential bias. To focus specifically on OF outcomes within the similar setting and mitigate selection bias related to baseline severity differences influencing outcomes, we restricted the Xiangya cohort to patients without mild AP patients, because the MIMIC-IV database is an ICU database which could reduce the selection bias. This deliberate restriction was intended to align the validation cohort with the target population for which the model was designed: patients with sufficient severity to warrant ICU-level care or close monitoring. Consequently, the model is intended for early risk stratification within ICU or high-dependency settings, rather than for general emergency department triage where mild cases predominate. The target population is therefore patients with AP judged to require ICU-level care, high-dependency care or close monitoring. Neither the performance reported here nor the 0.2 threshold has been evaluated in consecutive AP admissions that include mild disease. The identification of AP patients and the outcomes in the MIMIC-IV database (1429 patients) and Xiangya cohort (n = 216) involved referencing the International Classification of Diseases (ICD-9/10) codes. The classification of AP were based on the criteria of revised Atlanta classification [4]. Criteria of Aetiology are listed as follows: (1) biliary: choledocholithiasis or cholelithiasis by enhanced computed tomography or ultrasound; (2) hypertriglyceridaemia: triglyceride level > 1000 mg/dl; (3) alcoholic: drinking > 50 g/d for at least 1 year. This study follows Transparent Reporting of a Multivariable Prediction Model for Individual Prognosis or Diagnosis (TRIPOD+AI) guidelines [18]. The TyG index, one of the most interesting variables for this study, was defined as: $TyG=\text{ln}\left(\frac{triglycerides\;(\frac{mg}{dL})\times glucose(\frac{mg}{dL})}{\text{2}}\right)$ [10]. Because MIMIC-IV is an ICU database in which patients are seldom in a defined fasting state, we used the first triglyceride and glucose values recorded within 24 h of admission. These are generally non-fasting, stress-influenced measurements, so the resulting TyG index should be interpreted as an admission-based approximation rather than a strictly fasting index. The mortality was calculated as the crude mortality in this study due to the retrospective nature. The workflow, including patient selection in both discovery and validation datasets, model development, and validation procedures, is shown in Fig 1.

### Handling of missing data

The proportion of missing observations for every candidate predictor in each cohort is reported in S2 Table. No missingness threshold was applied and all candidate variables were retained. The MIMIC-IV development cohort and the Xiangya evaluation cohort were imputed separately, so no information was transferred between cohorts. Imputation used multiple imputation by chained equations with predictive mean matching (mice package), with five imputed datasets and 50 iterations each [19]. The outcome variable was included in the imputation model. Imputation was performed on each cohort before resampling rather than within resampling folds, and a single completed dataset was carried forward to feature selection, model fitting and external evaluation. The same imputation procedure was applied in both cohorts. A complete-case sensitivity analysis was not performed and is noted as a limitation.

### Feature selection and model development

As LASSO regularization can be sensitive to collinear variables, Pearson correlation filtering was performed to identify and remove highly correlated features such that no pair exceeded a correlation threshold (r > 0.7), thus addressing multicollinearity issues before the feature selection among the discovery dataset. The Boruta algorithm and LASSO were used to identify the most important features by comparing the Z-value of each feature against that of “shadow features” and L1 regularization, respectively. To derive a robust set of predictors, we applied both feature selection methods independently to the discovery cohort. We then retained only the intersection of features selected by both methods for subsequent model development.

Following feature selection, the key variables identified by both Boruta and LASSO algorithms, were used as the unified feature set for all subsequent models, representing the main families of machine-learning algorithms together with a neural-network benchmark, including five machine-learning algorithms: logistic regression (Elastic net), support vector machine, random forest, extreme gradient boosting (XGBoost), light gradient boosting machine, and a feedforward multilayer perceptron (MLP) as a neural-network benchmark. The MLP is a shallow architecture for static tabular data and does not stand in for deep-learning methods built for longitudinal or sequential EHR data. For consistency with the original analysis code, this model is labelled DNN within the figure panels; DNN and MLP refer to the same two-hidden-layer feedforward network throughout. For ML models, a benchmarking test involving nested resampling, automatic hyperparameter tuning and random search techniques was performed in the discovery set to optimize model performance by minimizing the Brier score (S1 Fig). The MLP model was trained by minimizing the binomial cross-entropy loss. Using adjustable weight parameters of the hidden layer, the MLP model was trained by the Adam optimizer with an initial learning rate of 0.0001. The number of modeling epochs was set to 1000 in this study. A 10-fold cross-validation repeated 20 times was applied in the discovery sets to prevent overfitting. It was also used to formulate the predictive models. Detailed hyperparameters of the final multilayer perceptron are provided in the S6 Table. During hyperparameter tuning, the model configuration achieving the lowest Brier Score was selected as optimal. The analysis proceeded in four steps. The original, non-updated models developed in MIMIC-IV were externally evaluated in the Xiangya cohort, and these are the primary external-evaluation results. Recalibration was anticipated because the model was developed in MIMIC-IV and applied to a cohort with a different case-mix and event rate. That cohort was then used to select the best-performing algorithm, to set the 0.2 operating threshold, and to fit logistic recalibration. The recalibrated model was assessed in the same cohort. Because selection, threshold setting and updating all took place in the cohort used for assessment, the estimates for the recalibrated deployment model are in-sample and require an additional independent cohort.

The discriminative performance in the validation set of each predictive model was assessed by receiver-operating characteristic curve (ROC) area under the curve (AUC). All classification metrics were computed at a probability threshold of 0.2 in the external validation cohort. Accuracy, precision, recall and F1 were derived from the confusion matrices (S2 Fig). With 45 events available, 95% confidence intervals for sensitivity, specificity, positive and negative predictive value, accuracy, F1 and Brier score at the 0.2 threshold were obtained by non-parametric bootstrap with 4000 resamples of the evaluation cohort, using the percentile method, applied to the untransformed predicted probabilities. Brier score, log loss, calibration slope and calibration intercept in Table 3 correspond to the original models applied to the Xiangya cohort. Brier score and log loss after logistic recalibration are given in S3 Table. Calibration slope and intercept are not reported for the recalibrated models, because recalibration was fitted in the same cohort and these parameters are therefore fixed at 1 and 0 by construction. Calibration, defined as the agreement between predicted probabilities and observed outcomes, was assessed using calibration plots and quantified by the calibration intercept and slope. To assess clinical utility a decision curve analysis (DCA) was performed plotting net benefit curves for each model and compared to the net benefit of ‘treat all’ and ‘treat none’ strategies [20]. The final model was developed based on the optimal model and feature set above. To determine the optimal risk threshold for the final model in our validation cohort, we generated a clinical impact curve (CIC) on the final model which could rigorously assess the most effective decision threshold for clinical application [21]. The x-axis of CIC showed different risk thresholds and their corresponding net benefit values, while the y-axis illustrated the number of positive patients identified by the model versus the actual true positives in a sample of all the validation dataset individuals. To improve calibration, logistic recalibration of each model was performed in the validation cohort; the decision-curve and clinical-impact analyses used the recalibrated probabilities, whereas the discrimination, classification metrics and Brier score reported correspond to the raw (non-updated) models [22]. We employed logistic regression to model the relationship between BISAP scores and new-onset OF.

### Model explanation

Other than predicting new-onset OF following AP, another important aim of this study was to identify the most important factors and their contribution to the prediction model using SHAP which uses Shapley values to explain how the predicted outcome for an observation is affected by particular feature values, in both ML and MLP models [23]. SHAP did not only provide validation of our feature selection approach, but also additional insights beyond what feature selection alone can offer, including individual-level explanations. An open-access Shiny application was built as a research prototype from the recalibrated model with the highest ROC-AUC, returning a risk estimate and an individual explanation. Four clinicians from the participating institutions gave informal feedback on functionality and face validity. This was not a usability study, workflow-integration study, prospective simulation or clinical-impact evaluation, and no usability instrument, task-completion metric or clinician decision outcome was recorded.

### Statistical analysis

The Mann-Whitney U test was used for continuous variables, and categorical variables were expressed as percentages (%) and Pearson chi-squared tests were used to compare differences between OF and non-OF group. Using the SHAP method, a beeswarm plot was drawn to show the contribution of each feature to the prediction results. SHAP evaluations of the validation datasets revealed how much a feature affected individual predictions to help understand the model’s decision-making process. All models were implemented using the ‘tidymodels’ suite of R packages [24]. No samples were overlapping at the patient level in the discovery and validation data sets. All statistical analyses were performed in R software (version: 4.3.3) and p-values< 0.05 (two-sided) were considered significant.

## Results

### Baseline characteristics and outcomes in the discovery and validation dataset

Table 1 shows the clinical characteristics of the 1429 AP patients enrolled in the discovery dataset. The mean age was 61.6 ± 17.2 years and 812 (56.8%) were male. The overall 28-day mortality was 11.8%. Notably, patients who developed new-onset OF had a significantly higher 28-day mortality (24.3% vs. 7.8%, p < 0.001) and experienced longer hospital and ICU stays. Laboratory and vital signs assessments on the first day of admission revealed that AP patients with new-onset OF had significantly higher levels of white blood cells (WBC), platelets, blood urea nitrogen (BUN), chloride, lactate dehydrogenase and TyG index. Conversely, they demonstrated lower albumin and bicarbonate compared to patients without OF. Similarly, there were 216 AP patients enrolled in the validation cohort during the 4-year-study period (Table 2). The Aetiology of 216 patients was categorized as biliary (n = 83, 38.4%), hypertriglyceridaemia (n = 76, 35.2%), alcoholic (n = 11, 5.1%) and other causes (n = 46, 21.3%). The mean age of the patients was 48.2 years. 149 (69%) and 67 (31%) patients were male and female, respectively. Forty-five patients met the criteria for new-onset OF within 28 days after admission.

**Table 1:** Comparison of baseline characteristics between non-OF and OF groups in the MIMIC-IV discovery cohort.

	Total	Non-OF	OF	*P* value
	*N = 1429*	*N = 1088*	*N = 341*	
Age, years (mean ± SD)	61.6 ± 17.2	61.7 ± 17.3	61.6 ± 16.7	0.932
Gender, n (%)				0.473
Female	617 (43.2)	476 (43.8)	141 (41.3)	
Male	812 (56.8)	612 (56.2)	200 (58.7)	
Hospitalization, days (mean±SD)	14.1 ± 15.0	11.5 ± 12.2	22.4 ± 19.4	<0.001
ICU stays, days (mean±SD)	5.05 ± 8.28	3.23 ± 4.39	10.9 ± 13.5	<0.001
Clinical characteristic on the admission day				
Hematocrit, %	35.0 ± 6.42	34.9 ± 6.19	35.1 ± 7.11	0.734
Hemoglobin	11.5 ± 2.24	11.5 ± 2.18	11.5 ± 2.42	0.998
Platelets count, 10^3^/mm^3^	236 ± 133	230 ± 127	254 ± 148	0.008
WBC count, 10^3^/mm^3^	14.6 ± 8.62	13.6 ± 7.83	17.8 ± 10.1	<0.001
Albumin, g/dL	3.22 ± 0.69	3.30 ± 0.67	2.99 ± 0.70	<0.001
Aniongap, mmol/L	16.9 ± 5.29	16.7 ± 5.32	17.5 ± 5.15	0.015
Bicarbonate, mmol/L	23.8 ± 4.02	23.9 ± 3.85	23.2 ± 4.47	0.004
BUN, mg/dL	27.7 ± 22.7	25.3 ± 21.3	35.2 ± 25.3	<0.001
Calcium, mg/dL	8.44 ± 0.80	8.46 ± 0.78	8.37 ± 0.86	0.080
Chloride, mmol/L	105 ± 5.75	105 ± 5.54	106 ± 6.29	0.003
Sodium, mmol/L	139 ± 4.57	139 ± 4.29	140 ± 5.34	0.054
Potassium, mmol/L	4.49 ± 0.79	4.45 ± 0.78	4.62 ± 0.80	<0.001
Basophils count, 10^3^/mm^3^	0.03 ± 0.04	0.03 ± 0.04	0.04 ± 0.05	0.751
Eosinophils count, 10^3^/mm^3^	0.12 ± 0.76	0.10 ± 0.14	0.19 ± 1.53	0.277
Lymphocytes count, 10^3^/mm^3^	1.38 ± 1.56	1.37 ± 1.62	1.39 ± 1.39	0.814
Monocytes count, 10^3^/mm^3^	0.71 ± 0.54	0.68 ± 0.52	0.81 ± 0.58	<0.001
Neutrophils count, 10^3^/mm^3^	11.4 ± 7.71	10.6 ± 7.23	13.9 ± 8.62	<0.001
Inr	1.60 ± 0.96	1.55 ± 0.88	1.77 ± 1.15	0.002
Pt, second	17.6 ± 10.5	17.0 ± 9.35	19.5 ± 13.3	0.001
PTT, second	39.7 ± 23.3	38.1 ± 21.6	44.8 ± 27.5	<0.001
Alt, U/L	174 ± 636	150 ± 541	250 ± 867	0.044
Alp, U/L	160 ± 170	156 ± 152	171 ± 218	0.224
Ast, U/L	304 ± 1297	231 ± 959	534 ± 2014	0.008
Bilirubin total, mg/dL	2.44 ± 4.66	2.24 ± 4.17	3.11 ± 5.91	0.012
Lactate dehydrogenase, U/L	497 ± 859	450 ± 743	644 ± 1144	0.003
TyG index	8.80 ± 0.78	8.76 ± 0.77	8.90 ± 0.78	0.004
Outcome, %				<0.001
Survival	1261 (88.2)	1003 (92.2)	258 (75.7)	
Death	168 (11.8)	85 (7.8)	83 (24.3)	

Abbreviations: OF: organ failure; TyG: Triglyceride-glucose; BUN, blood urea nitrogen; WBC, white blood cells; PTT, Partial thromboplastin time

**Table 2:** Comparison of baseline characteristics between non-OF and OF groups in the Xiangya validation cohorts.

	Total	Non-OF	OF	*P* value
	*N = 216*	*N = 171*	*N = 45*	
Age, years (mean ± SD)	48.2 ± 13.0	48.3 ± 13.2	48.1 ± 12.3	0.945
Gender, n (%)				0.106
Female	67 (31.0)	58 (33.9)	9 (20.0)	
Male	149 (69.0)	113 (66.1)	36 (80.0)	
Aetiology, n (%)				0.826
Biliary	83 (38.4)	67 (39.2)	16 (35.6)	
Alcoholism	11 (5.1)	9 (5.26)	2 (4.44)	
Hypertriglyceridaemia	76 (35.2)	57 (33.3)	19 (42.2)	
Others	46 (21.3)	38 (22.1)	8 (17.8)	
ICU stays, days (mean±SD)	12.9 ± 16.5	9.67 ± 13.8	25.1 ± 20.1	<0.001
Hospitalization, days (mean±SD)	37.6 ± 25.1	37.3 ± 22.5	38.7 ± 33.7	0.784
Clinical characteristic on the admission day, (mean ± SD)				
Aniongap, mmol/L	14.5 + 2.46	14.5 + 2.40	14.3 + 2.70	0.594
Bicarbonate, mmol/L	22.5 + 3.11	22.5 + 3.21	22.7 + 2.75	0.643
Calcium, mg/dL	8.05 + 0.61	8.04 + 0.64	8.09 + 0.45	0.552
Chloride, mmol/L	103 + 3.83	103 + 3.80	103 + 3.95	0.629
Sodium, mmol/L	138 + 3.07	138 + 2.98	138 + 3.38	0.380
Potassium, mmol/L	4.19 + 0.43	4.18 + 0.41	4.22 + 0.49	0.573
Basophils count, 10^3^/mm^3^	0.02 + 0.02	0.02 + 0.02	0.02 + 0.02	0.904
Eosinophils count, 10^3^/mm^3^	0.03 + 0.04	0.03 + 0.04	0.04 + 0.05	0.470
Monocytes count, 10^3^/mm^3^	0.64 + 0.47	0.63 + 0.46	0.65 + 0.51	0.839
Inr	1.17 + 0.30	1.16 + 0.31	1.22 + 0.28	0.220
Pt, second	13.0 + 3.35	12.9 + 3.42	13.5 + 3.04	0.200
PTT, second	29.1 + 7.98	29.2 + 7.99	29.1 + 8.00	0.969
Alt, U/L	36.1 + 22.6	36.0 + 22.0	36.4 + 25.2	0.933
Alp, U/L	96.9 + 41.2	95.1 + 39.7	104.0 + 46.3	0.243
Ast, U/L	49.7 + 28.8	49.5 + 28.3	50.5 + 30.9	0.852
Bilirubin total, μmol/L	6.01 + 2.58	6.21 + 2.51	5.27 + 2.73	0.042
Lactate dehydrogenase, U/L	241.7 + 78.7	247.3 + 77.2	220.5 + 81.6	0.052
BUN, mmol/L	10.4 + 10.8	7.07 + 4.76	22.9 + 16.8	<0.001
Albumin, g/L	30.0 + 5.70	30.5 + 6.00	27.9 + 3.75	<0.001
Platelets count, 10^3^/mm^3^	222 + 116	234 + 119	176 + 88.0	<0.001
Lymphocytes count, 10^3^/mm^3^	0.98 + 0.64	0.98 + 0.58	1.00 + 0.84	0.863
WBC count, 10^3^/mm^3^	12.7 + 8.82	12.3 + 8.73	14.1 + 9.12	0.252
Neutrophils count, 10^3^/mm^3^	10.3 + 6.26	9.93 + 5.48	11.7 + 8.54	0.204
Hematocrit, %	28.1 + 6.39	28.7 + 6.40	26.0 + 5.93	0.010
TyG index	9.41 + 0.97	9.28 + 0.96	9.90 + 0.88	<0.001
Highest temperature, °C	38.6 + 1.04	38.6 + 1.02)	38.6 + 1.10)	0.691
Outcome, %				<0.001
Survival	164 (75.9)	153 (89.5)	11 (24.4)	
Death	52 (24.1)	18 (10.5)	34 (75.6)	

Abbreviations: OF: organ failure; TyG: Triglyceride-glucose; BUN, blood urea nitrogen; WBC, white blood cells; PTT, Partial thromboplastin time.

### Feature selection and model development

**Fig 2:**
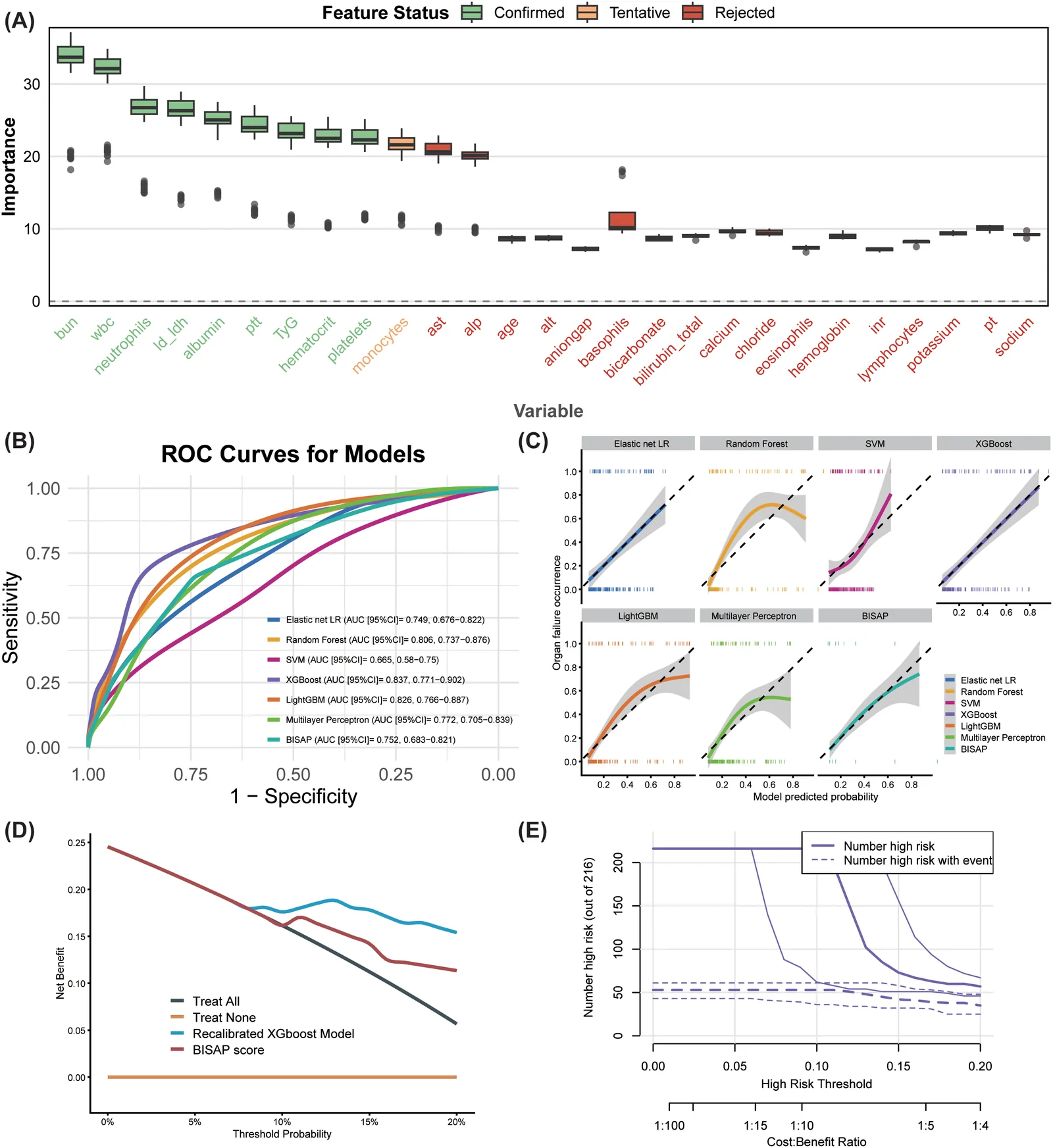
A Feature selection based on Boruta algorithm. The horizontal axis represents the name of each characteristic, and the vertical axis represents the Z-score for each variable. The box plot illustrates the Z-score distribution of each variable during model computation. Green boxes represent important variables identified by the model, while red boxes indicate unimportant variables. Blue box represents the magnitude of the Z-Score. The yellow box represents tentative variables; B. Receiver operating characteristic curves for the machine learning models; C. Calibration curve for the recalibrated machine learning models; D. Decision curve analysis of recalibrated XGBoost model and BISAP score; E. Clinical Impact Curve of recalibrated XGBoost model in the validation cohort; Abbreviations: LR: logistic regression; XGBoost: extreme gradient boosting; SVM: support vector machine; LightGBM: gradient boosting machine; AUC: area under the curve.

Boruta and lasso regression were used to screen the relevant features of the discovery dataset. Using LASSO method with 10-fold cross validation, 16 out of 27 characteristics were selected for the model construction with the coefficients (S3 Fig). Nine characteristics were confirmed as important (Fig 2A) using the Boruta algorithm, of which 6 were consistent with the results obtained from LASSO analysis. The 6 variables which closely associated with new-onset OF were platelets, albumin, bun, WBC, partial thromboplastin time (PTT) and TyG index.

We constructed five machine-learning models and one multilayer-perceptron benchmark to estimate the risk of new-onset OF in AP patients in the discovery cohort. Boosted tree models had the best performance and discrimination for new-onset OF, with the XGBoost model exhibiting the highest performance with a ROC AUC of 0.837 [95% confidence interval (CI): 0.771 − 0.902] in the validation dataset (Fig 2B). The light gradient boosting machine closely followed (AUC [95%CI]= 0.826, 0.766 − 0.887) and random forest (AUC [95%CI]= 0.806, 0.737 − 0.876) showed comparable discriminative efficacy and outperformed the remaining algorithms. The remaining models, while still demonstrating good predictive power, ranked as follows in descending order of performance: MLP (AUC [95%CI]= 0.772, 0.705 − 0.839), BISAP score (AUC [95%CI]= 0.752, 0.683 − 0.821), Elastic net logistic regression (AUC [95%CI]= 0.749, 0.676 − 0.822) and SVM (AUC [95%CI]= 0.665, 0.58 − 0.75). On external validation, the original XGBoost model retained strong discrimination but, as expected for a model transported to a different case-mix, showed a calibration slope below 1 (Table 3), indicating overly extreme predictions. Logistic recalibration was performed on all models to achieve method parity with the test of the BISAP algorithm. Recalibration maintained the same ROC-AUC as this is a rank-based measure unaffected by monotonic transformations (S4 Fig). Details of other performance metrics for the 6 raw models and BISAP score are shown in Table 3. Calibration of the original models is shown in S5 Fig and calibration after logistic recalibration in Fig 2C. Recalibration improved calibration for all six models, and XGBoost and elastic net logistic regression had the lowest Brier score and log loss after updating (S3 Table). Among the original models, BISAP was the best calibrated (Table 3). Logistic recalibration, applied as a model-updating step, corrected this and was used for the deployed application (S3 Table). DCA of clinical utility revealed that the recalibrated boosted tree models yielded consistently superior net benefit across clinically-relevant risk thresholds (Figs 2D and S6). BISAP also demonstrated superior clinical utility over ‘treat all’ and ‘treat none’ strategies at risk thresholds exceeding 10%, but XGBoost proved superior still across a broader range of thresholds from 10% in the DCA. XGBoost had the best discrimination and ranked among the best models for calibration and clinical utility, so it was selected as the final model (S6–S8 Fig). This selection was made in the Xiangya cohort, so the performance of the selected model carries selection made in the same data and is not independent external validation of the deployed model. The recalibration did not change the AUC-ROC, but the XGBoost model demonstrated the highest AUC and was among the best-calibrated models after recalibration (S3 Table). Detailed parameters of the XGBoost model are given in S4 Table. Bootstrap 95% confidence intervals for all candidate models and BISAP at the 0.2 threshold are given in S5 Table. Detailed parameters of the multilayer perceptron are given in S6 Table, and the confusion matrices underlying the classification metrics. Intervals spanned roughly 0.3 for sensitivity, positive predictive value and F1, so threshold-dependent performance in this cohort is estimated imprecisely and should be read as indicative.

**Table 3:** Performance of the original (non-updated) candidate models and the BISAP score on external evaluation in the Xiangya cohort.

Model	Accuracy	Precision	Recall	F1 Score	Brier Score	Log Loss	Calibration Slope	Calibration Intercept
**LR**	0.537	0.248	0.600	0.351	0.161	0.499	0.393	-0.840
**SVM**	0.537	0.257	0.644	0.367	0.163	0.507	0.375	-0.893
**XGB**	0.690	0.347	0.556	0.427	0.160	0.496	0.300	-0.956
**RF**	0.532	0.259	0.667	0.373	0.160	0.497	0.311	-0.905
**MLP**	0.565	0.275	0.667	0.390	0.159	0.494	0.440	-0.791
**LightGBM**	0.616	0.302	0.644	0.411	0.159	0.494	0.291	-0.945
**BISAP**	0.667	0.325	0.556	0.410	0.153	0.478	0.624	-0.446

Abbreviations: LR, Logistic Regression; RF, Random Forest; SVM, Support Vector Machine; XGBoost, eXtreme Gradient Boosting; LightGBM, Light Gradient Boosting Machine; MLP, Multilayer Perceptron; BISAP: Bedside Index for Severity in Acute Pancreatitis

To evaluate classification performance across risk thresholds, clinical impact curves were constructed for the XGBoost model, comparing the number of patients labelled high risk with the number of true positives at increasing decision thresholds. Curves are shown for the recalibrated model, which is the version deployed in the application (Fig 2E), and for the original non-updated model (S8 Fig). For example, a high-risk threshold of 0 corresponds to inappropriately considering all patients as high risk. In the evaluation cohort, the original non-updated model at a threshold of 0.2 identified 25 of the 45 patients who developed OF and produced 47 false positives (S7 Table), so this threshold favours case detection over precision. Because logistic recalibration shifts predicted probabilities, the counts obtained at a fixed 0.2 threshold differ between the original and the recalibrated model, and the classification metrics reported throughout refer to the original models.

### Interpretability analysis

**Fig 3:**
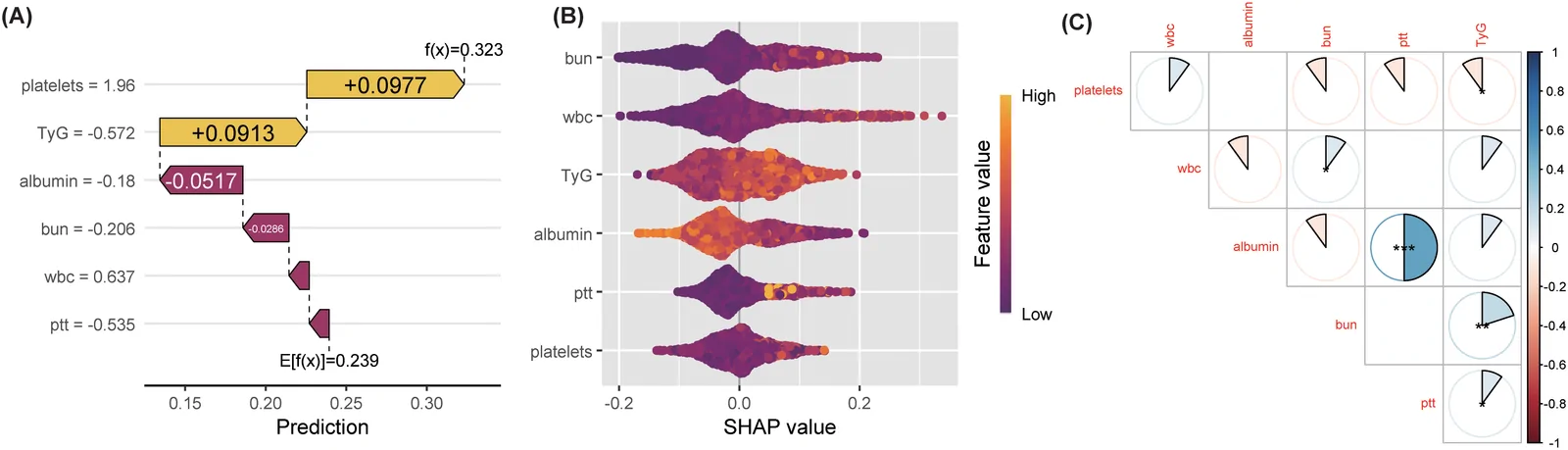
SHAP-based interpretation of the XGBoost model in the evaluation cohort. A. SHAP force plot; B. SHAP summary plot; C. Correlation heatmap plot of most important variables with their SHAP value. Abbreviations: BUN: blood urea nitrogen; TyG: triglyceride glucose index; PTT: Partial thromboplastin time; WBC: white blood cells.

To assess the contribution of each clinical variable to the model’s predictions we calculated SHAP scores for each prediction. Fig 3A shows the force plot for an individual’s predicted risk of OF, with yellow features representing positive predictors and red features indicating negative predictors. For this specific patient, our XGBoost model predicted a higher risk of OF compared to the baseline risk (i.e., the proportion of cases with OF in the validation cohort). Fig 3B illustrates the variables’ contributions to predictions for all individuals in the validation set. For example, for many individuals higher albumin concentrations resulted in lower predictions of risk of OF. Platelets, TyG index, albumin, BUN, WBC and PTT emerged as the 6 most important factors in predicting OF. Fig 3C shows the Pearson’s correlation heatmap of SHAP values for the above 6 continuous variables which underwent data scaled and indicated the significant correlation between these 6 variables with SHAP values.

### Application of model

The selected model, trained in MIMIC-IV and recalibrated in the Xiangya cohort, is available as an open-access web application (https://wudikdfz.shinyapps.io/Decentapp). It takes the six admission-day variables and returns an estimated probability of new-onset OF with SHAP values for each variable. The application is a research prototype for risk calculation, not a validated decision-support system, and its interface states that it was evaluated only in patients without mild AP.

## Discussion

In this study, we developed machine-learning models and evaluated them externally in a severity-enriched cohort. XGBoost discriminated new-onset OF better than the other algorithms evaluated and better than the BISAP score, using clinical data collected within the first 24 h of admission. After recalibration, XGBoost retained the highest AUC, and its Brier score was among the lowest of the recalibrated models. To provide improved interpretability of the model, SHAP analysis showed the contribution of platelets, TyG index, albumin, BUN, WBC and PTT to the model.

Recent years have seen a surge in AI-driven prognostic models for OF in AP [14]. For example, Shi et al. developed a nomogram-based model using clinical variables, achieving an AUC of 0.814 [7], while Chen et al. used a convolutional neural network to analyze CT images, and reported a notably higher AUC of 0.920 [11]. On this static tabular dataset, and among the algorithms evaluated, the tree-based models outperformed the MLP benchmark, in keeping with reports that tree-based methods remain competitive on tabular data [25]. The comparison does not extend to architectures built for imaging or for longitudinal EHR sequences, which we did not test. Similarly to Kui et al. who constructed an XGBoost model for prediction and obtained AUC values of 0.81, our XGBoost model achieved a higher AUC [26]. Although simple comparison of the AUC values is difficult due to differences in cohort characteristics, race, and so on, we supply the code demonstrating the prediction tool’s development and assessment for reproducibility.

Langmead et al. have used a novel 5-cytokine panel to construct a random forest model for the prediction of severity with AUC up to 0.89 in the cohort where the discovery dataset (n = 60) was smaller than validation dataset (n = 133) [27]. Whilst a novel cytokine panel could shed light on the mechanism of the cytokine storm, it has limited clinical availability due to not being routinely measured. Thapa et al. built three prognostic models for severe AP following an alternative severity classification system instead of revised Atlanta classification, and found that the XGBoost model was better than the MLP model with a high hidden layer. Our results are highly consistent with theirs, though again they do not share their code which restricts opportunities for validation by other investigators [28]. In recent years, machine-learning and neural-network models have been widely used in the medical field, showing strong performance in other clinical areas from cardiovascular disease to skin cancer [23,29]. Compared to the regularized logistic regression algorithm, we found the tree-based ML models can effectively capture non-linear relationships and build the final model by integrating several weak classifiers, thus resulting in better generalization ability which is robust compared to noisy data. Whilst the AUC of our XGBoost model did not surpass some of the published models, we have used open-access database and a clear definition to train models thus reducing the risk of bias, and have made the code available, addressing a key reporting gap identified by Critelli et al. who found that over 90% of studies lacked such transparency in their recent systematic review [14].

ML and MLP models are generally seen as “black boxes” [11]. Indeed, data are processed through complex layers of computation, and it is difficult to identify the most relevant features for sample classification and limited with the lack of interpretability [30]. SHAP values could help to understand the contribution of important features, especially platelets, BUN and TyG index, and reveal feature-specific impact mechanisms in the optimal prediction model. Consistent with the previous study, we considered platelet as the principal predictor in the prediction model of OF which may correlate with OF and reflect underlying comorbidities or the systemic inflammatory response [31]. In line with previous studies, elevated BUN has been consistently associated with severe AP patients in the intensive care setting with acute kidney injury which may not only be a marker of advanced renal dysfunction but also may indicate the combined deterioration of cardiac and renal function [2]. Consistent with Wang et al., we observed that TyG index was a predictor for OF which has been validated previously as an independent prognostic biomarker for high-risk of all-cause mortality in critically ill patients [10]. TyG index may impact AP patients through complex mechanisms interacting with IR which is a chronic, low-grade systemic inflammation characterized by high circulating levels of pro-inflammatory cytokines [32]. The inflammatory cascade accompanying metabolic dysregulation and IR potentiates immune hyperactivation, exacerbating pancreatic acinar cell injury [33]. There is a biologically plausible mechanism underlying the association between the TyG index and OF among AP patients. Meng et al. have shown that early glycemic control (i.e., in the first 72 hours post-admission) is associated with the severity of AP, because stress response may exacerbate IR due to ongoing inflammatory processes, thereby, complicating glucose regulation [34]. There is also a strong relationship between ectopic fat, especially intrapancreatic fat deposition, and pancreatic injury [35]. Ectopic fat, which is ascribed to IR, activates pro-inflammatory molecules such as interleukin-6, and those molecules could play a critical role in the pathogenesis of AP in patients with IR [32]. However, the exact mechanism of the causal relationship among IR, OF, and AP has not been fully elucidated [36,37]. Our finding that the TyG index (a marker of IR) is a significant predictor of OF supports the hypothesis that underlying IR may contribute to the pathophysiology linking metabolic dysfunction to OF in AP. The apparently conflicting results regarding the correlation between diabetes and severity of AP presented in past studies could be resolved by further investigation through multi-omics approaches [36].

We compared the predictive ability of six models and the BISAP score for new-onset OF using ROC-AUC and other performance metrics. The XGBoost model showed the best discrimination among those evaluated. In addition, because the characteristics we incorporated from an open-access database had commonly used clinical indicators, they are easy to measure and implement in future studies. We have made the XGBoost model available as a free web-based application that returns the predicted probability of OF together with an explanation for the individual prediction. The application is a research prototype and has not been evaluated within a clinical workflow.

The proposed computational framework delivers dual clinical utilities: 1) prognostic stratification: clinicians may identify patients at higher estimated risk on admission. Whether this should change monitoring intensity or organ-support decisions was not tested here. For example, TyG index is a risk factor for OF. This could be due to comorbidities which may become worse due to AP and contribute to a poor outcome and have not been directly related to any other organ dysfunction, giving possible personalized care for these special populations at admission. On the other hand, our study observed that a substantial proportion of non-OF patients were admitted to the ICU for a mean length of stay exceeding 3 days. The favourable discrimination of our model is consistent with the possibility of identifying such patients. This raises the possibility that risk stratification could inform ICU triage. It is a hypothesis from a retrospective analysis: we did not observe, and could not assess, any change in ICU admissions, costs or complications, which would require prospective clinical-impact and health-economic evaluation. 2) patient communication: our online tool with SHAP value can enhance prognostic transparency and shared decision-making of prognosis and doctor-patient communication. Our SHAP analysis makes the risk estimate interpretable and may help in explaining risk to patients. We did not test whether management guided by the model changes outcomes. Prospective usability, workflow-integration, clinical-impact and health-economic studies are needed before use in routine care.

Despite the novel findings of our study, several limitations to the study need to be acknowledged. Firstly, this model was developed using an open-access and retrospective cohort, MIMIC-IV, over an extensive time span with an external validation in two Chinese hospitals. The MIMIC-IV cohort was older, had lower 28-day mortality, and a different etiological profile, with hypertriglyceridaemia accounting for 35.2% of cases in the Xiangya cohort. Furthermore, patients with mild AP were excluded from the Xiangya cohort to align the severity distribution between cohorts. As a result, the model was evaluated in a severity-enriched population; consequently, the reported performance may not transfer directly to consecutive admissions that include mild cases. The proposed 0.2 risk threshold may therefore overestimate risk when applied to broader, unselected populations. The model has not been evaluated in unselected admissions that include mild AP, and should not be used for general emergency-department triage until it has. The external validation cohort contained 45 organ-failure events for a six-predictor model. At this ratio, threshold-based classification metrics are inherently unstable and sensitive to the chosen operating threshold, whereas threshold-independent measures, such as AUC and the Brier score, are more robust. The classification metrics in Table 3 should therefore be read as indicative rather than definitive, and larger validation cohorts are needed for precise estimates. Nonetheless, our deployment of a web-based interface gives an opportunity for others to validate this tool which over 90% of existing models have failed to provide. Future studies may also introduce variability in clinical management practices and data collection due to evolving guidelines, potentially leading to heterogeneous effects on clinical outcomes and impacting the model’s applicability. Missing admission-day values were imputed rather than excluded, a single completed dataset was carried forward rather than pooled across imputations, and a complete-case sensitivity analysis was not performed; bias cannot be excluded if values were not missing at random. Secondly, the importance of TyG index suggests that replacing blood glucose levels with the TyG index in the prognostic scoring systems could improve their performance. However, this study used an open-access database which did not examine the time span between IR and AP nor whether patients had underlying IR prior to pancreatic injury, resulting in beta-cell dysfunction and consequent IR. As our clinical features have only been captured in the first 24h, further studies should perform analysis on the temporal variability or dynamic modeling. Thirdly, gene or protein expression data were not available in the screening of predictors for OF prediction due to the retrospective nature of the MIMIC database, although such data could enhance the understanding for the pathological mechanism and improve the performance of model in various contexts. Future studies focusing on integrating large-scale biobank databases, such as FinnGen and UK Biobank, may leverage advanced AI, developing high-quality models providing more insights into the mechanism of OF. Fourthly, while the combination of ML models and interpretable SHAP algorithm facilitate clinician decisions, it is crucial to acknowledge that clinical judgment cannot be entirely replaced by AI in the era of precision medicine now. With 45 events, the evaluation cohort could not support reliable subgroup analysis, and we judged unstable stratified estimates to be less informative than none. Discrimination and calibration were therefore not adequately assessed within strata of age, sex, Aetiology or comorbidity, and fairness across demographic and clinical subgroups has not been established. Transportability across aetiologies is also uncertain, since hypertriglyceridaemia accounted for 35.2% of the Xiangya cohort but is uncommon in biliary- or alcohol-predominant populations. Subgroup and fairness assessment will need substantially larger independent cohorts. Model selection, threshold selection and logistic recalibration were all carried out in the Xiangya cohort. Calibration of the updated model is therefore an in-sample estimate, and the deployed model needs evaluation in a further independent cohort before any claim of external validity. The neural network was a two-hidden-layer feedforward MLP applied to static admission-day variables. It is a reasonable benchmark for that data structure but not a proxy for recurrent or transformer architectures built for longitudinal EHR data, which the absence of temporal features precluded. Our conclusions apply to the algorithms and the static tabular data evaluated here. In addition, because MIMIC-IV records routine ICU laboratory values rather than protocolized fasting samples, the glucose component of the TyG index was non-fasting in most patients. Although non-fasting TyG indices have retained prognostic value in prior critically ill cohorts, this measurement characteristic introduces variability, and prospective validation using standardized fasting samples is warranted. Finally, prospective international or large multi-center consortiums with efforts to build a generalizable model, following TRIPOD+AI reporting guideline, have been lacking, which risks siloing attempts at building predictive models, so extending collaborations within follow-up studies could ensure further reproducibility and generalizability.

## Conclusions

An interpretable XGBoost model discriminated new-onset OF well on external evaluation in a multicentre cohort of AP patients requiring ICU-level, high-dependency or close monitoring. Model selection, threshold selection and recalibration all used that cohort, so the deployed model requires a further independent cohort, and its behaviour in unselected admissions including mild AP is unknown. The Shiny application is a research prototype; prospective usability, workflow-integration, clinical-impact and health-economic studies are needed before use in routine care.

## Supporting information

S1 Fig Hyperparameter tuning results of (A) Logistic Regression model, (B) Random Forest model, (C) Support Vector Machine model, (D) eXtreme Gradient Boosting model, (E) Light Gradient Boosting Machine model and (F) Multilayer Perceptron model in the discovery cohort. MLP is labelled DNN in the Fig panels; both refer to the same two-hidden-layer feedforward network.
(PDF)

S2 Fig Confusion Matrix ground truth showing the accuracy of (A) Logistic Regression model, (B) Random Forest model, (C) Support Vector Machine model, (D)eXtreme Gradient Boosting model, (E) Light Gradient Boosting Machine model, (F) Multilayer Perceptron model and (G) BISAP score in the validation cohorts’ classification for predicting organ failure. MLP is labelled DNN in the Fig panels; both refer to the same two-hidden-layer feedforward network.
(PDF)

S3 Fig Feature selection: (A) Variation characteristics of variable coefficients in the Lasso algorithm; (B) The process of selecting the optimal value of the parameter λ in the lasso algorithm is carried out by the cross-validation method. (PDF)

S4 Fig Receiver operating characteristic curves for the recalibrated machine learning models. MLP is labelled DNN in the Fig panels; both refer to the same two-hidden-layer feedforward network.
(PDF)

S5 Fig Calibration curve of all the raw machine learning models. MLP is labelled DNN in the Fig panels; both refer to the same two-hidden-layer feedforward network.
(PDF)

S6 Fig Decision curve analysis of all the recalibrated models and BISAP score. MLP is labelled DNN in the Fig panels; both refer to the same two-hidden-layer feedforward network.
(PDF)

S7 Fig Decision curve analysis of all the raw models and BISAP score. MLP is labelled DNN in the Fig panels; both refer to the same two-hidden-layer feedforward network.
(PDF)

S8 Fig Clinical Impact Curve of raw XGBoost model in the validation cohort. (PDF)

S1 Table TRIPOD-AI checklist. (PDF)

S2 Table Missing observations for each candidate predictor in the MIMIC-IV development cohort and the Xiangya evaluation cohort. (DOCX)

S3 Table Brier score and log loss of the recalibrated machine-learning models and the multilayer-perceptron benchmark in the Xiangya evaluation cohort. (DOCX)

S4 Table The parameters of XGBoost model and update recalibration. (DOCX)

S5 Table Threshold-dependent performance of the original (non-updated) candidate models and the BISAP score at the 0.2 risk threshold in the Xiangya evaluation cohort, with bootstrap 95% confidence intervals. (DOCX)

S6 Table The configuration of MLP model. (DOCX)
