# Supplementary material for: Development and external evaluation of an interpretable machine-learning model for early prediction of organ failure in higher-risk acute pancreatitis patients: A multicentre cohort study
Source: PLOS Digit Health. 2026 Sep 22;5(9):e0001735. doi: 10.1371/journal.pdig.0001735 (PMC13596832; doi:10.1371/journal.pdig.0001735)
Supplement: Supplementary file 1 [file pdig.0001735.s001.pdf]

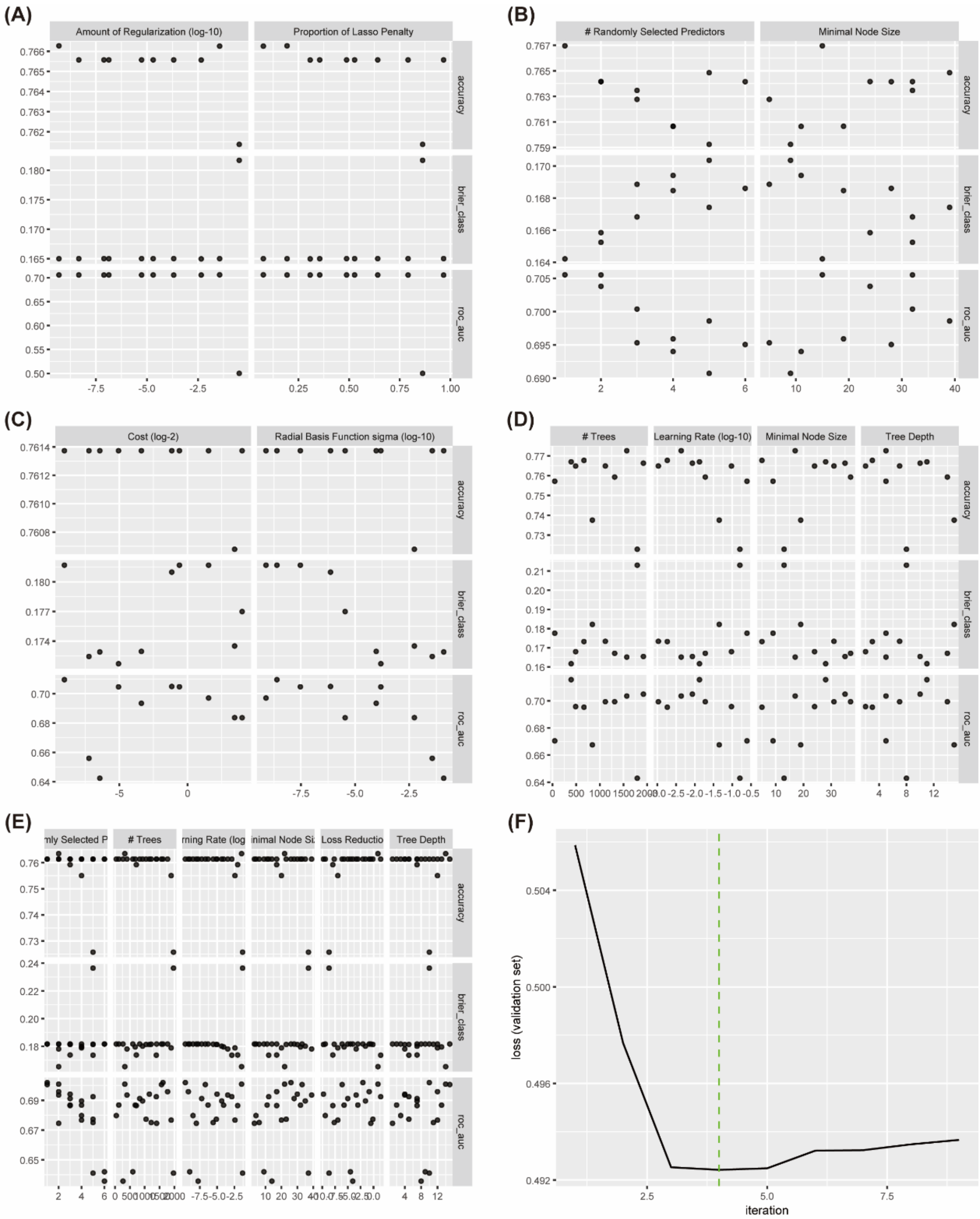

(A)
Amount of Regularization (log-10)
Proportion of Lasso Penalty
accuracy
brier_class
roc_auc
(B)
# Randomly Selected Predictors
Minimal Node Size
(C)
Cost (log-2)
Radial Basis Function sigma (log-10)
(D)
# Trees
Learning Rate (log-10)
Minimal Node Size
Tree Depth
(E)
# Trees
Tree Depth
(F)
loss (validation set)
iteration
