# Supplementary material for: Development and external evaluation of an interpretable machine-learning model for early prediction of organ failure in higher-risk acute pancreatitis patients: A multicentre cohort study
Source: PLOS Digit Health. 2026 Sep 22;5(9):e0001735. doi: 10.1371/journal.pdig.0001735 (PMC13596832; doi:10.1371/journal.pdig.0001735)
Supplement: Supplementary file 2 [file pdig.0001735.s002.pdf]

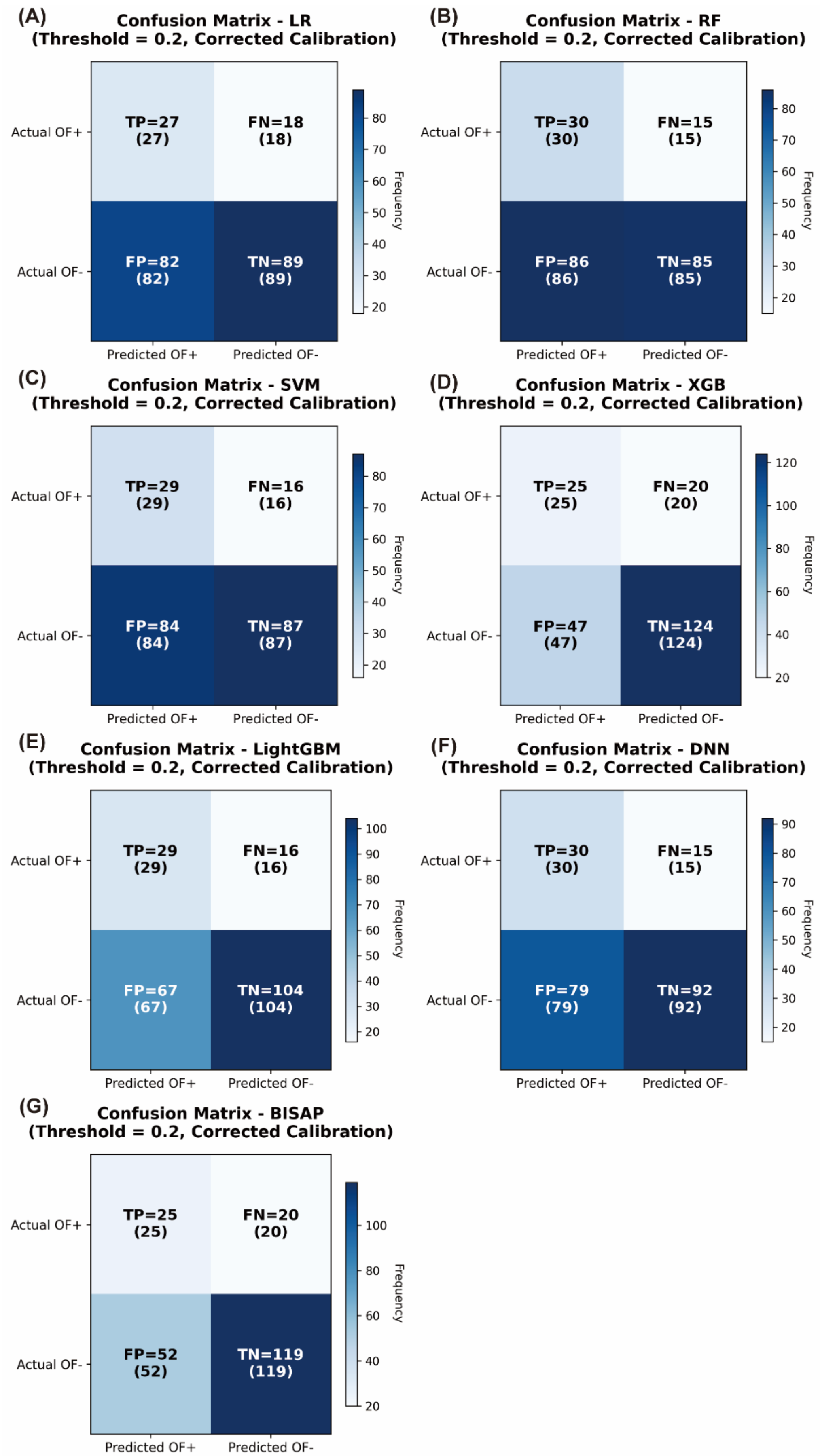
(A)
Confusion Matrix - LR
(Threshold = 0.2, Corrected Calibration)
Actual OF+
Actual OF-
TP=27 (27)
FN=18 (18)
FP=82 (82)
TN=89 (89)
Predicted OF+
Predicted OF-
Frequency
80
70
60
50
40
30
20
(B)
Confusion Matrix - RF
(Threshold = 0.2, Corrected Calibration)
Actual OF+
Actual OF-
TP=30 (30)
FN=15 (15)
FP=86 (86)
TN=85 (85)
Predicted OF+
Predicted OF-
Frequency
80
70
60
50
40
30
20
(C)
Confusion Matrix - SVM
(Threshold = 0.2, Corrected Calibration)
Actual OF+
Actual OF-
TP=29 (29)
FN=16 (16)
FP=84 (84)
TN=87 (87)
Predicted OF+
Predicted OF-
Frequency
80
70
60
50
40
30
20
(D)
Confusion Matrix - XGB
(Threshold = 0.2, Corrected Calibration)
Actual OF+
Actual OF-
TP=25 (25)
FN=20 (20)
FP=47 (47)
TN=124 (124)
Predicted OF+
Predicted OF-
Frequency
120
100
80
60
40
20
(E)
Confusion Matrix - LightGBM
(Threshold = 0.2, Corrected Calibration)
Actual OF+
Actual OF-
TP=29 (29)
FN=16 (16)
FP=67 (67)
TN=104 (104)
Predicted OF+
Predicted OF-
Frequency
100
90
80
70
60
50
40
30
20
(F)
Confusion Matrix - DNN
(Threshold = 0.2, Corrected Calibration)
Actual OF+
Actual OF-
TP=30 (30)
FN=15 (15)
FP=79 (79)
TN=92 (92)
Predicted OF+
Predicted OF-
Frequency
90
80
70
60
50
40
30
20
(G)
Confusion Matrix - BISAP
(Threshold = 0.2, Corrected Calibration)
Actual OF+
Actual OF-
TP=25 (25)
FN=20 (20)
FP=52 (52)
TN=119 (119)
Predicted OF+
Predicted OF-
Frequency
100
80
60
40
20
