# Supplementary material for: Development and external evaluation of an interpretable machine-learning model for early prediction of organ failure in higher-risk acute pancreatitis patients: A multicentre cohort study
Source: PLOS Digit Health. 2026 Sep 22;5(9):e0001735. doi: 10.1371/journal.pdig.0001735 (PMC13596832; doi:10.1371/journal.pdig.0001735)
Supplement: Supplementary file 3 [file pdig.0001735.s003.pdf]

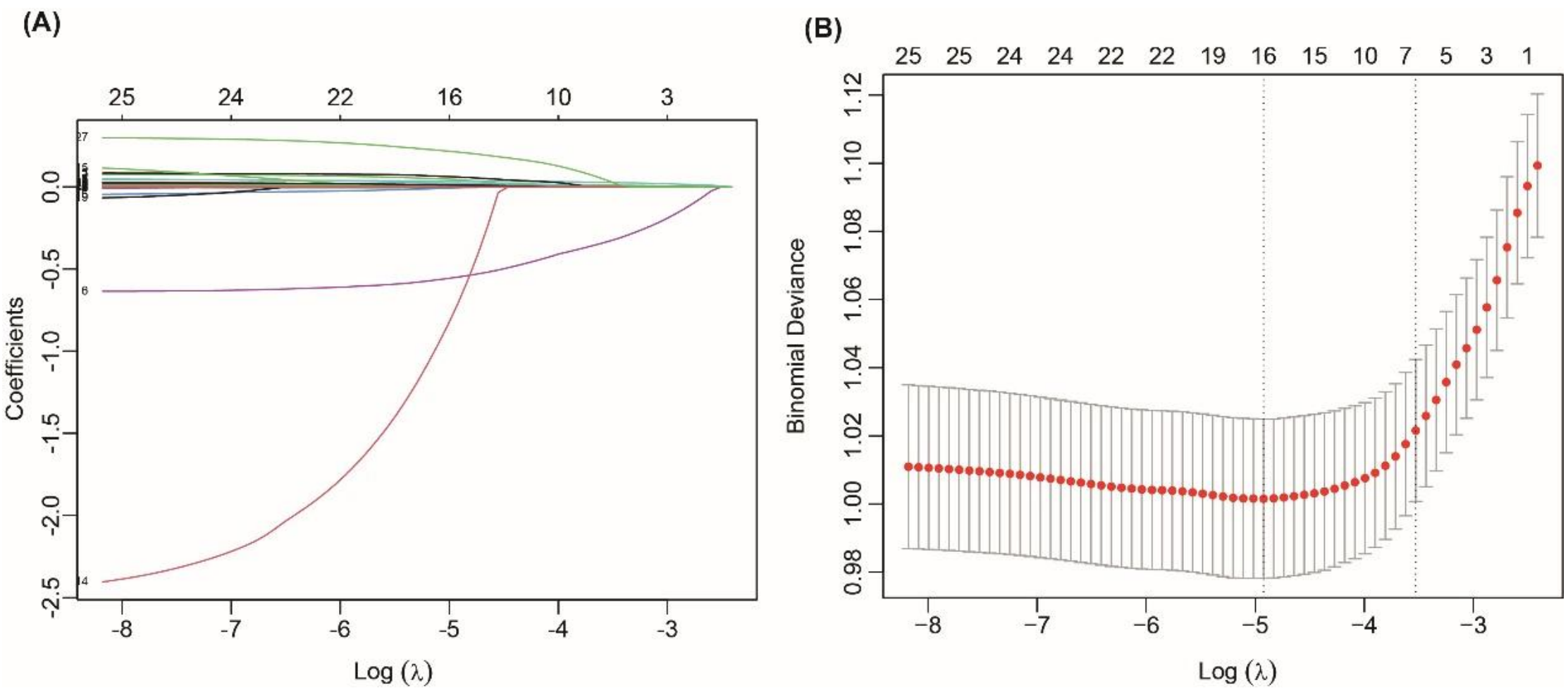
(A)
25
24
22
16
10
3
Coefficients
0.0
-0.5
-1.0
-1.5
-2.0
-2.5
-8
-7
-6
-5
-4
-3
Log (λ)
(B)
25
25
24
24
22
22
19
16
15
10
7
5
3
1
Binomial Deviance
1.12
1.10
1.08
1.06
1.04
1.02
1.00
0.98
-8
-7
-6
-5
-4
-3
Log (λ)
