# Supplementary material for: Development and external evaluation of an interpretable machine-learning model for early prediction of organ failure in higher-risk acute pancreatitis patients: A multicentre cohort study
Source: PLOS Digit Health. 2026 Sep 22;5(9):e0001735. doi: 10.1371/journal.pdig.0001735 (PMC13596832; doi:10.1371/journal.pdig.0001735)
Supplement: Supplementary file 4 [file pdig.0001735.s004.pdf]

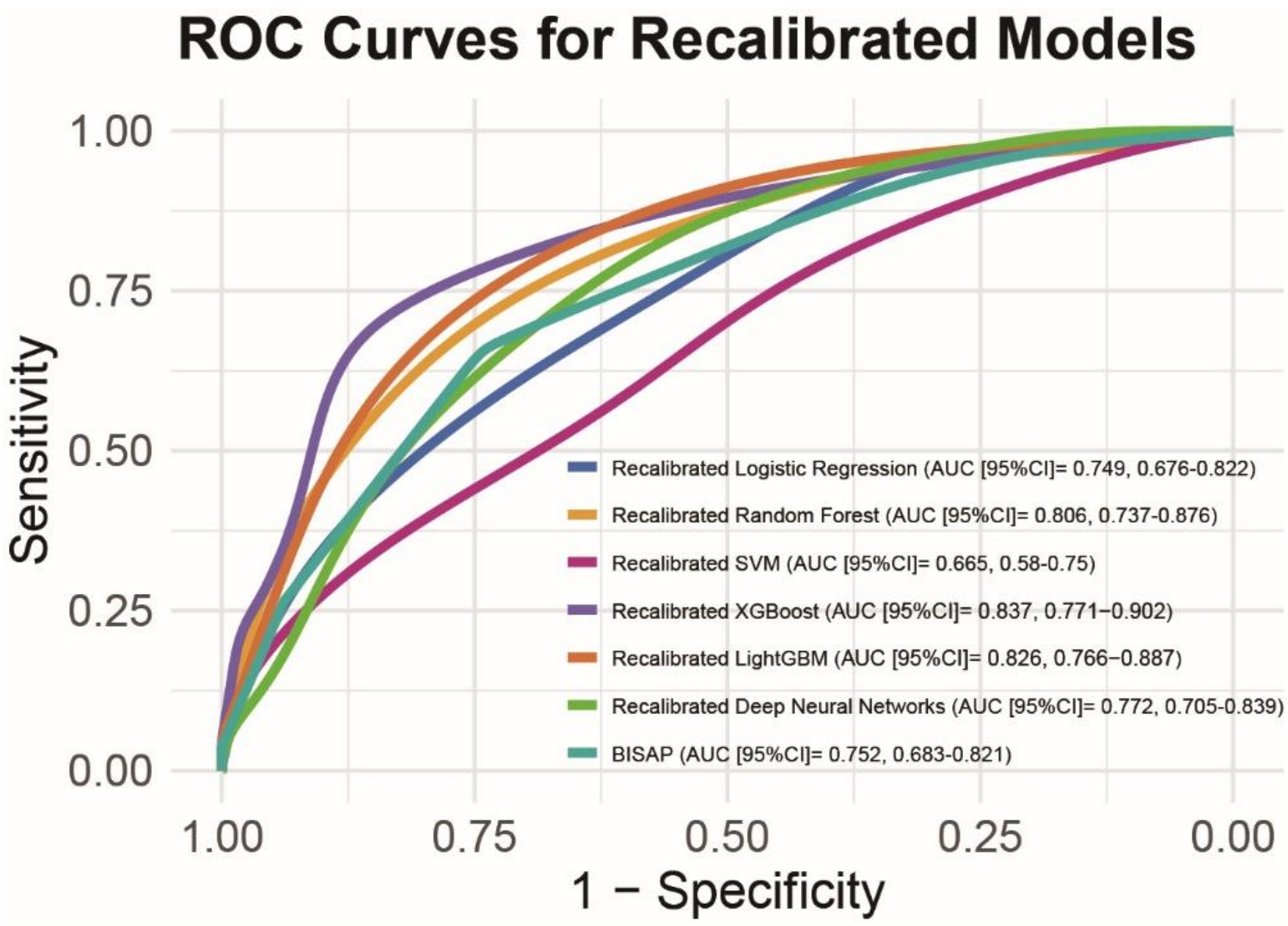
ROC Curves for Recalibrated Models
1.00
0.75
0.50
0.25
0.00
Sensitivity
Recalibrated Logistic Regression (AUC [95%CI]= 0.749, 0.676-0.822)
Recalibrated Random Forest (AUC [95%CI]= 0.806, 0.737-0.876)
Recalibrated SVM (AUC [95%CI]= 0.665, 0.58-0.75)
Recalibrated XGBoost (AUC [95%CI]= 0.837, 0.771−0.902)
Recalibrated LightGBM (AUC [95%CI]= 0.826, 0.766−0.887)
Recalibrated Deep Neural Networks (AUC [95%CI]= 0.772, 0.705-0.839)
BISAP (AUC [95%CI]= 0.752, 0.683-0.821)
1.00
0.75
0.50
0.25
0.00
1 − Specificity
