# Supplementary material for: Development and external evaluation of an interpretable machine-learning model for early prediction of organ failure in higher-risk acute pancreatitis patients: A multicentre cohort study
Source: PLOS Digit Health. 2026 Sep 22;5(9):e0001735. doi: 10.1371/journal.pdig.0001735 (PMC13596832; doi:10.1371/journal.pdig.0001735)
Supplement: Supplementary file 5 [file pdig.0001735.s005.pdf]

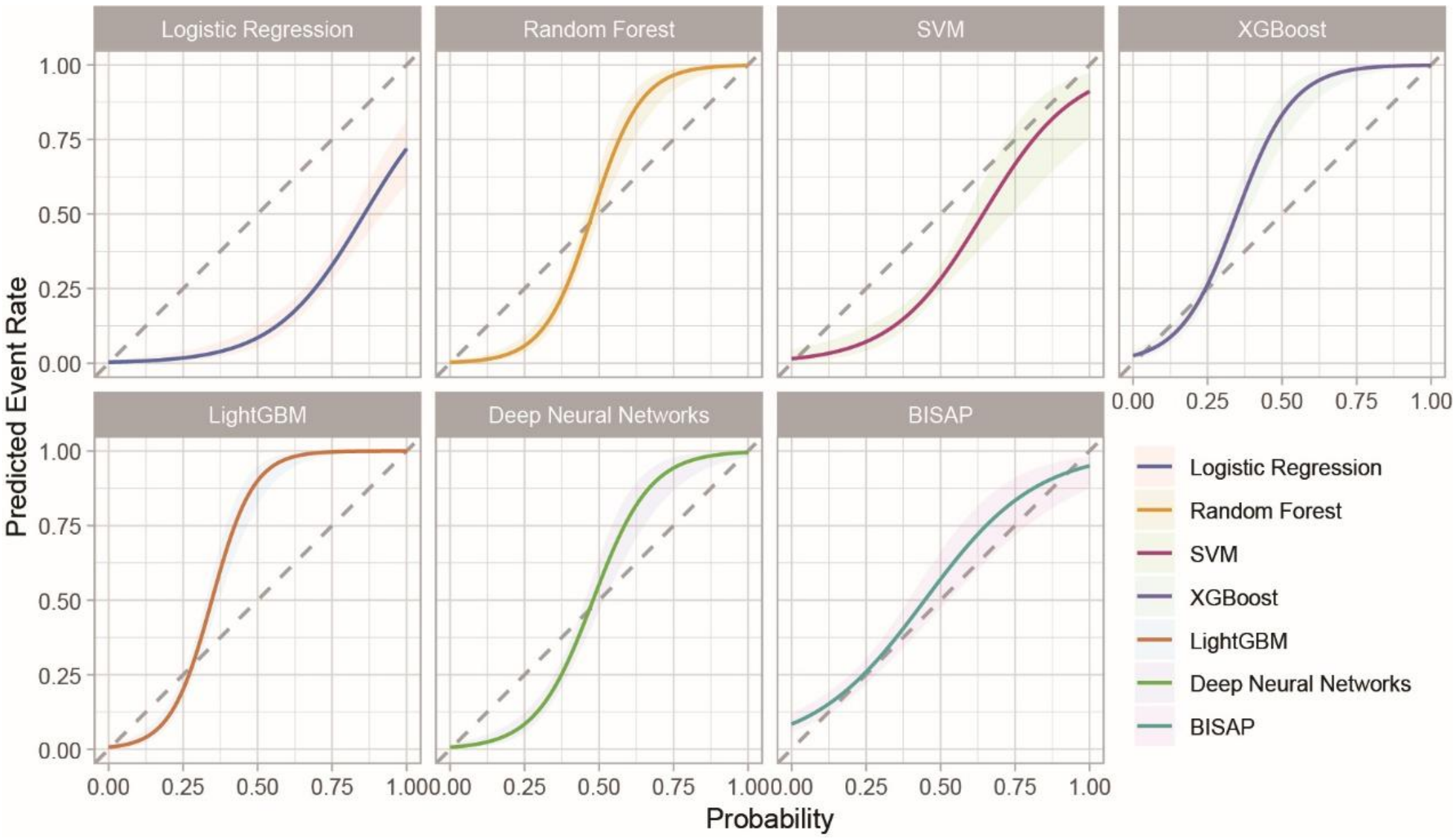

Logistic Regression
Random Forest
SVM
XGBoost
LightGBM
Deep Neural Networks
BISAP
Predicted Event Rate
Probability
0.00
0.25
0.50
0.75
1.00
Logistic Regression
Random Forest
SVM
XGBoost
LightGBM
Deep Neural Networks
BISAP
