# Supplementary material for: Development and external evaluation of an interpretable machine-learning model for early prediction of organ failure in higher-risk acute pancreatitis patients: A multicentre cohort study
Source: PLOS Digit Health. 2026 Sep 22;5(9):e0001735. doi: 10.1371/journal.pdig.0001735 (PMC13596832; doi:10.1371/journal.pdig.0001735)
Supplement: Supplementary file 7 [file pdig.0001735.s007.pdf]

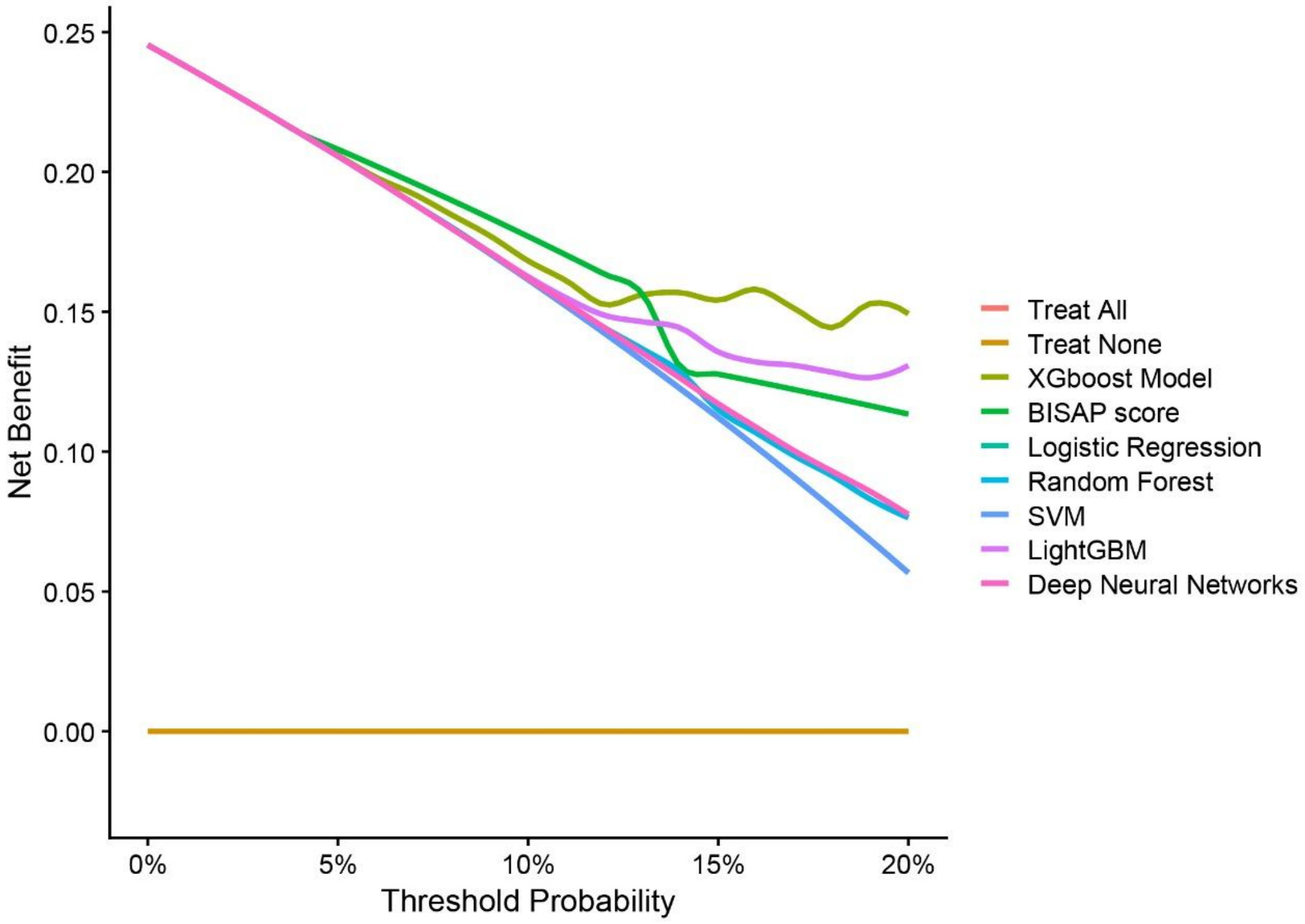

0.25
0.20
0.15
0.10
0.05
0.00
Net Benefit
0%
5%
10%
15%
20%
Threshold Probability
Treat All
Treat None
XGboost Model
BISAP score
Logistic Regression
Random Forest
SVM
LightGBM
Deep Neural Networks
