# Supplementary material for: Development and external evaluation of an interpretable machine-learning model for early prediction of organ failure in higher-risk acute pancreatitis patients: A multicentre cohort study
Source: PLOS Digit Health. 2026 Sep 22;5(9):e0001735. doi: 10.1371/journal.pdig.0001735 (PMC13596832; doi:10.1371/journal.pdig.0001735)
Supplement: Supplementary file 8 [file pdig.0001735.s008.pdf]

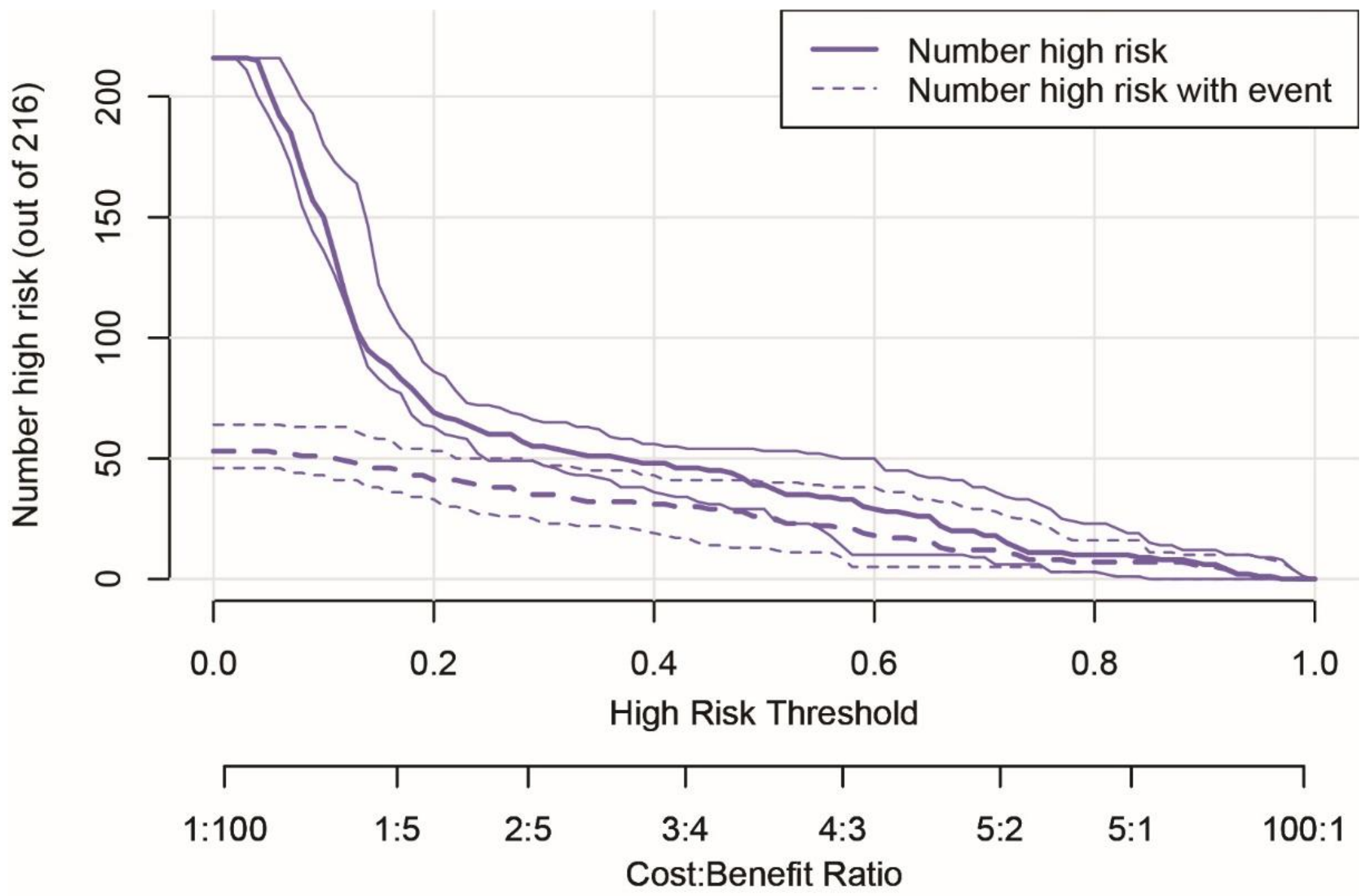

Number high risk
Number high risk with event
Number high risk (out of 216)
0
50
100
150
200
0.0
0.2
0.4
0.6
0.8
1.0
High Risk Threshold
1:100
1:5
2:5
3:4
4:3
5:2
5:1
100:1
Cost:Benefit Ratio
