# Supplementary material for: Development and external evaluation of an interpretable machine-learning model for early prediction of organ failure in higher-risk acute pancreatitis patients: A multicentre cohort study
Source: PLOS Digit Health. 2026 Sep 22;5(9):e0001735. doi: 10.1371/journal.pdig.0001735 (PMC13596832; doi:10.1371/journal.pdig.0001735)
Supplement: Supplementary file 10 [file pdig.0001735.s010.docx]

**S2 Table Missing observations for each candidate predictor in the MIMIC-IV development cohort and the Xiangya evaluation cohort.**

| **Variable** | **MIMIC-IV development cohort (n = 1429)** | | **Xiangya evaluation cohort (n = 216)** | |
| --- | --- | --- | --- | --- |
|  | **n missing** | **% missing** | **n missing** | **% missing** |
| Haematocrit, % | 209 | 14.6 | 12 | 5.6 |
| Haemoglobin, g/dL | 130 | 9.1 | 18 | 8.3 |
| Platelet count, 10^3^/mm^3^† | 229 | 16.0 | 33 | 15.3 |
| White blood cell count, 10^3^/mm^3^† | 263 | 18.4 | 14 | 6.5 |
| Albumin, g/dL† | 161 | 11.3 | 12 | 5.6 |
| Anion gap, mmol/L | 119 | 8.3 | 27 | 12.5 |
| Bicarbonate, mmol/L | 77 | 5.4 | 17 | 7.9 |
| Blood urea nitrogen, mmol/L† | 210 | 14.7 | 29 | 13.4 |
| Calcium, mg/dL | 119 | 8.3 | 30 | 13.9 |
| Chloride, mg/dL | 244 | 17.1 | 11 | 5.1 |
| Creatinine, mg/dL | 244 | 17.1 | 33 | 15.3 |
| Glucose, mg/dL† | 144 | 10.1 | 16 | 7.4 |
| Sodium, mg/dL | 277 | 19.4 | 22 | 10.2 |
| Potassium, mg/dL | 91 | 6.4 | 14 | 6.5 |
| Basophil count, 10^3^/mm^3^ | 253 | 17.7 | 30 | 13.9 |
| Eosinophil count, 10^3^/mm^3^ | 244 | 17.1 | 34 | 15.7 |
| Lymphocyte count, 10^3^/mm^3^ | 186 | 13.0 | 42 | 19.4 |
| Monocyte count, 10^3^/mm^3^ | 153 | 10.7 | 29 | 13.4 |
| Neutrophil count, 10^3^/mm^3^ | 249 | 17.4 | 31 | 14.4 |
| International normalised ratio | 256 | 17.9 | 30 | 13.9 |
| Prothrombin time, s | 223 | 15.6 | 12 | 5.6 |
| Partial thromboplastin time, s† | 120 | 8.4 | 20 | 9.3 |
| Alanine aminotransferase, U/L | 89 | 6.2 | 18 | 8.3 |
| Alkaline phosphatase, U/L | 93 | 6.5 | 20 | 9.3 |
| Aspartate aminotransferase, U/L | 207 | 14.5 | 23 | 10.6 |
| Total bilirubin, μmol/L | 151 | 10.6 | 17 | 7.9 |
| Lactate dehydrogenase, U/L | 129 | 9.0 | 41 | 19.0 |
| Triglycerides, mg/dL† | 210 | 14.7 | 30 | 13.9 |
| Age, years | 0 | 0.0 | 0 | 0.0 |

**†** Variable contributing to the six final predictors. The triglyceride–glucose index was derived from triglycerides and glucose, so both source variables are marked.

All values refer to the admission-day measurements used for model development and evaluation. No missingness threshold was applied and all candidate variables were retained. The two cohorts were imputed separately using multiple imputation by chained equations with predictive mean matching (mice package), with five imputed datasets and 50 iterations each; the outcome variable was included in the imputation model. Among the variables contributing to the six final predictors, missingness did not exceed 18.4% in either cohort. Table S2 reports missingness for all admission-day variables extracted from each cohort. The candidate set entering feature selection was derived from these variables after the triglyceride and glucose measurements were combined into the triglyceride–glucose index and correlated variables were removed, as described in the Methods.

Abbreviations: MIMIC-IV, Medical Information Mart for Intensive Care IV.
