# Supplementary material for: Development and external evaluation of an interpretable machine-learning model for early prediction of organ failure in higher-risk acute pancreatitis patients: A multicentre cohort study
Source: PLOS Digit Health. 2026 Sep 22;5(9):e0001735. doi: 10.1371/journal.pdig.0001735 (PMC13596832; doi:10.1371/journal.pdig.0001735)
Supplement: Supplementary file 11 [file pdig.0001735.s011.docx]

**S3 Table Brier score and log loss of the recalibrated machine-learning models and the multilayer-perceptron benchmark in the Xiangya evaluation cohort**

| Model | Brier Score | Log Loss |
| --- | --- | --- |
| LR | 0.158 | 0.481 |
| RF | 0.140 | 0.440 |
| SVM | 0.172 | 0.524 |
| XGB | 0.131 | 0.409 |
| LightGBM | 0.130 | 0.404 |
| MLP | 0.157 | 0.471 |

Abbr: LR, Logistic Regression; RF, Random Forest; SVM, Support Vector Machine; XGBoost, eXtreme Gradient Boosting; LightGBM, Light Gradient Boosting Machine; MLP, Multilayer Perceptron

Values are in-sample, because logistic recalibration was fitted in the cohort in which it was assessed. Calibration slope and intercept are not shown for the same reason: fitting recalibration in this cohort constrains them to 1 and 0. The BISAP score is not included here; its performance before updating is given in Table 3.
