# Supplementary material for: Development and external evaluation of an interpretable machine-learning model for early prediction of organ failure in higher-risk acute pancreatitis patients: A multicentre cohort study
Source: PLOS Digit Health. 2026 Sep 22;5(9):e0001735. doi: 10.1371/journal.pdig.0001735 (PMC13596832; doi:10.1371/journal.pdig.0001735)
Supplement: Supplementary file 12 [file pdig.0001735.s012.docx]

**S4 Table The parameters of XGBoost model and update recalibration**

| **Name of parameters** | **Value** |
| --- | --- |
| Learning Rate | 0.0133178450949 |
| Maximum Depth | 11 |
| Minimum Loss Reduction | 0 |
| Column Sample by Tree | 1 |
| Column Sample by Node | 1 |
| Minimum Child Weight | 28 |
| Subsample Ratio | 1 |
| Number of Threads | 1 |
| Objective Function | binary |
| Number of Rounds | 394 |
| Number of Features | 6 |
| Intercept of logistic recalibration | -2.863 |
