# Supplementary material for: Development and external evaluation of an interpretable machine-learning model for early prediction of organ failure in higher-risk acute pancreatitis patients: A multicentre cohort study
Source: PLOS Digit Health. 2026 Sep 22;5(9):e0001735. doi: 10.1371/journal.pdig.0001735 (PMC13596832; doi:10.1371/journal.pdig.0001735)
Supplement: Supplementary file 13 [file pdig.0001735.s013.docx]

**S5 Table Threshold-dependent performance of the original (non-updated) candidate models and the BISAP score at the 0.2 risk threshold in the Xiangya evaluation cohort, with bootstrap 95% confidence intervals.**

| **Model** | **Sensitivity** | **Specificity** | **PPV** | **NPV** | **Accuracy** | **F1 score** | **Brier score** |
| --- | --- | --- | --- | --- | --- | --- | --- |
| Elastic net LR | 0.600 (0.455 to 0.741) | 0.520 (0.448 to 0.596) | 0.248 (0.168 to 0.333) | 0.832 (0.755 to 0.899) | 0.537 (0.472 to 0.602) | 0.351 (0.250 to 0.447) | 0.161 (0.131 to 0.193) |
| SVM | 0.644 (0.500 to 0.780) | 0.509 (0.435 to 0.583) | 0.257 (0.179 to 0.341) | 0.845 (0.770 to 0.909) | 0.537 (0.472 to 0.602) | 0.367 (0.268 to 0.462) | 0.163 (0.133 to 0.197) |
| Random forest | 0.667 (0.522 to 0.811) | 0.497 (0.424 to 0.571) | 0.259 (0.178 to 0.345) | 0.850 (0.774 to 0.919) | 0.532 (0.463 to 0.597) | 0.373 (0.270 to 0.471) | 0.160 (0.130 to 0.192) |
| MLP | 0.667 (0.522 to 0.810) | 0.538 (0.466 to 0.612) | 0.275 (0.193 to 0.364) | 0.860 (0.790 to 0.925) | 0.565 (0.500 to 0.634) | 0.390 (0.286 to 0.488) | 0.159 (0.129 to 0.192) |
| LightGBM | 0.644 (0.500 to 0.786) | 0.608 (0.536 to 0.680) | 0.302 (0.211 to 0.398) | 0.867 (0.802 to 0.926) | 0.616 (0.546 to 0.676) | 0.411 (0.303 to 0.510) | 0.159 (0.130 to 0.192) |
| **XGBoost** | 0.556 (0.409 to 0.702) | 0.725 (0.657 to 0.793) | 0.347 (0.239 to 0.460) | 0.861 (0.800 to 0.917) | 0.690 (0.625 to 0.750) | 0.427 (0.309 to 0.534) | 0.160 (0.130 to 0.193) |
| **BISAP** | 0.556 (0.407 to 0.700) | 0.696 (0.623 to 0.764) | 0.325 (0.224 to 0.432) | 0.856 (0.794 to 0.912) | 0.667 (0.602 to 0.727) | 0.410 (0.296 to 0.519) | 0.153 (0.124 to 0.185) |

Confidence intervals were obtained by non-parametric bootstrap with 4000 resamples of the evaluation cohort. All estimates were calculated from the untransformed (non-recalibrated) predicted probabilities. Point estimates were derived from the confusion-matrix counts in Table S7 and correspond to the values reported in Table 3 of the main text. No formal statistical comparison between models was undertaken, as the study was neither designed nor powered for that purpose.

Abbreviations: BISAP, Bedside Index for Severity in Acute Pancreatitis; LightGBM, Light Gradient Boosting Machine; LR, logistic regression; MLP, multilayer perceptron; NPV, negative predictive value; PPV, positive predictive value; SVM, support vector machine; XGBoost, eXtreme Gradient Boosting.
