# Supplementary material for: Development and external evaluation of an interpretable machine-learning model for early prediction of organ failure in higher-risk acute pancreatitis patients: A multicentre cohort study
Source: PLOS Digit Health. 2026 Sep 22;5(9):e0001735. doi: 10.1371/journal.pdig.0001735 (PMC13596832; doi:10.1371/journal.pdig.0001735)
Supplement: Supplementary file 14 [file pdig.0001735.s014.docx]

**S6 Table The configuration of MLP model**

| Parameter | Value |
| --- | --- |
| **Architecture** | Input (6) → Hidden1 (32, ReLU) → Hidden2 (8, ReLU) → Output (1, Sigmoid) |
| **Activation function** | ReLU (hidden layers), Sigmoid (output layer) |
| **Regularization** | L2 regularization (penalty = 0.01) |
| **Optimizer** | Adam |
| **Learning rate** | 0.0001 |
| **Batch size** | 32 (default) |
| **Maximum epochs** | 1000 |
| **Early stopping** | 20% training data as validation set; patience = 50 (default) |
| **Loss function** | Binary cross‑entropy |
